# Bayesian analysis of zero inflated spatiotemporal HIV/TB child mortality data through the INLA and SPDE approaches: Applied to data observed between 1992 and 2010 in rural North East South Africa

**DOI:** 10.1016/j.jag.2012.04.001

**Published:** 2013-06

**Authors:** Eustasius Musenge, Tobias Freeman Chirwa, Kathleen Kahn, Penelope Vounatsou

**Affiliations:** a MRC/Wits Rural Public Health & Health Transitions Research Unit, School of Public Health, Faculty of Health Sciences, University of the Witwatersrand, Johannesburg, South Africa; b Biostatistics and Epidemiology Division, School of Public Health, Faculty of Health Sciences, University of the Witwatersrand, Johannesburg, South Africa; c Swiss Tropical and Public Health Institute, Basel, Switzerland; d Centre for Global Health Research, Umeå University, Umeå, Sweden; e INDEPTH Network, Accra, Ghana

**Keywords:** GMRF, Big “N”, Zero inflated, INLA SPDE, HIV/TB mortality, Spatiotemporal, Agincourt South Africa

## Abstract

Longitudinal mortality data with few deaths usually have problems of zero-inflation. This paper presents and applies two Bayesian models which cater for zero-inflation, spatial and temporal random effects. To reduce the computational burden experienced when a large number of geo-locations are treated as a Gaussian field (GF) we transformed the field to a Gaussian Markov Random Fields (GMRF) by triangulation. We then modelled the spatial random effects using the Stochastic Partial Differential Equations (SPDEs). Inference was done using a computationally efficient alternative to Markov chain Monte Carlo (MCMC) called Integrated Nested Laplace Approximation (INLA) suited for GMRF. The models were applied to data from 71,057 children aged 0 to under 10 years from rural north-east South Africa living in 15,703 households over the years 1992–2010. We found protective effects on HIV/TB mortality due to greater birth weight, older age and more antenatal clinic visits during pregnancy (adjusted RR (95% CI)): 0.73(0.53;0.99), 0.18(0.14;0.22) and 0.96(0.94;0.97) respectively. Therefore childhood HIV/TB mortality could be reduced if mothers are better catered for during pregnancy as this can reduce mother-to-child transmissions and contribute to improved birth weights. The INLA and SPDE approaches are computationally good alternatives in modelling large multilevel spatiotemporal GMRF data structures.

## Highlights

► South Africa's HIV/TB burden in children and modelling which results in mortality risk maps. ► Analyses of hierarchical spatiotemporal data with zero inflated outcomes. ► Resolving the “Big N” on Gaussian fields (GF) by converting to Gaussian Markov Random Fields (GMRF). ► Use of fast and accurate approximate Bayesian algorithms, i.e. INLA and SPDE in lieu of MCMC. ► Medical or natural assumptions driven modelling that informs further research and policy.

## 1 Introduction

Public Health data on mortality have been growing increasingly rich as more accurate information on “who”, “where” and “when” becomes available. These form hierarchical (multilevel) data structures which are correlated such that person-level (“who”) information can be repeated, geo-statistical (“where”) data often has spatial correlation and temporal (“when”) data can be auto-correlated. Classical statistical techniques are usually based on independent observations, but when applied to multilevel data structures they often underestimate the standard errors. As a result of this the statistical significance is overestimated leading to erroneous results and subsequent inferences (Cressie, 1993). This defeats the main goal in epidemiological analysis, which is to identify and quantify correctly any exposures, behaviours and characteristics that may modify a population's or individuals risk and use these to implement more appropriate interventions (Rose, 2001).

In modelling hierarchical data we can take into account spatial and temporal correlations by introducing in the model spatiotemporal random effects. Several other hurdles have to be overcome when modelling hierarchical mortality data such as: zero inflation when there is a greater proportion of non-occurrence for an outcome, handling large data structures, repeated measures and estimating many parameters rapidly and accurately. Bayesian techniques with the aid of the Markov chain Monte Carlo (MCMC) simulation methods have successfully overcome these hurdles and fit spatiotemporal random effects for reasonably sized geo-locations of Gaussian fields (GF) (Berliner et al., 2000; Gilks et al., 1996; Casella and Robert, 1999; Wikle, 2003; Wikle et al., 1998). However as the number of geo-locations increases, MCMC computations of a dense GF *m* × *m* spatial correlation matrix become infeasible or extremely slow in the order of power three (*O*(*m*^3^)), this problem is popularly known as the “big m” or “big N” (Banerjee et al., 2004). Several approaches have been used to resolve the “big m”. Banerjee et al. (2004) give brief summaries of these: sub-sampling, spectral, lattice, dimension reduction and course fine coupling methods (Banerjee et al., 2004, 2008; Banerjee and Carlin, 2003; Kamman and Wand, 2001; Johnson et al., 1990; French et al., 2002). Generally these techniques attempt to reduce the dimension of the GF by selecting a “representative” sub-sample or fixing some parameters or changing the scale from continuous to discrete with the aim of reducing the computational burden in running MCMC simulations.

We addressed this problem using techniques proposed by Rue and Held (2005) who changed the continuous scale GF to a discrete scale Gaussian Markov Random Field (GMRF), for the Matérn family of covariance structures (Rue and Held, 2005). More recently Lindgren et al. (2011) provides the detail of how the GF and GMRF relate via Stochastic Partial Differential Equations (SPDE) using basis functions (Lindgren et al., 2011; Cameletti et al., 2012). Secondly we performed inference and prediction using Integrated Nested Laplace Approximation (INLA) well suited for GMRF as opposed to the commonly used MCMC (Rue et al., 2009). Hence we greatly reduced the computational burden and could do in hours what usually took days having reduced the computational operations for a spatiotemporal model from power 3 to power 1.5 (*O*(*m*^3^) → *O*(*m*^3/2^)).

The aim of this paper is to discuss and apply a Bayesian model that can handle large zero-inflated spatiotemporal observational data on mortality producing reliable estimates speedily. In Section 2 we explore the Bayesian methods and model fitting inference, prediction and goodness of fit. Section 3 we apply the discussed Bayesian spatiotemporal model to the data from Agincourt in rural South Africa which has 71,057 children aged 0–9 years living in 15,703 households over the years 1992–2010. In Section 4 we discuss the merits of our model and distil the Public Health implications of our results in interventional studies.

## 2 Methods

### 2.1 Spatiotemporal model structure

The outcome *y*_*i*_(*s*_*j*_, *t*) was the observed HIV/TB related death of a child *i* = 1, …, *N* from a given household *j* = 1, …, *m* in a specific year *t* = 1, …, *T* which is a realisation of the spatio-temporal process *y*(. , .) ∈ *Y*(. , .). Assuming the outcomes distribution belongs to the exponential family of distributions, we can fit flexible structural additive models belonging to the generalized linear mixed models (GLMM) (Brezger and Lang, 2006; Fahrmeir and Lang, 2001). Our data may be represented by the equation:

(1) $$y_{i}(s_{j},t)=X(s_{j},t)\beta +\sum_{j=1}^{n_{f}}f^{\left(j\right)}(u_{ji},t)+\varepsilon_{i}(s_{j},t)$$

where *X*(*s*_*j*_, *t*) is the design matrix with fixed *p* covariates, ***β*** = (*β*_0_, … *β*_*p*_)′ is the regression coefficients vector, *f*(.) which is one of the *f*^(*j*)^ used to relax the linear relationship or introduce random effects or both and $\varepsilon_{i}(s_{j},t)∼N(0,\sigma_{\varepsilon }^{2})$ are the error terms which are neither temporally nor spatially correlated (Cameletti et al., 2012). As our data are spatially and temporally correlated we can introduce random effects *f*(.) = *f*(*s*_*j*_, *t*) a Gaussian random field with a first order autoregressive temporal effect *ξ*(*s*_*j*_, *t* − 1) and coefficient *ϕ* and zero mean multivariate normal (temporally independent) spatial effects $\omega (s_{j},t)∼MVN(0,\Sigma =\sigma_{\omega }^{2}C(||s_{j}-s_{k}||=h);j\neq k)$ resulting in the equation:

(2) $$f(s_{j},t)=\phi \xi (s_{j},t-1)+\omega (s_{j},t)$$

where |*ϕ*|<1 in case of stationarity, $\xi (s_{j},1)∼N(0,\sigma_{\omega }^{2}/(1-\phi^{2})=1/\tau_{\omega }(1-\phi^{2}))$ and the spatial effect is second order stationary and isotropic. When the spatial correlation follows a Matérn covariance structure we obtain *C*(*h*) = (1/(*Γ*(*ν*)2^*ν*−1^))(*κh*)^*ν*^*K*_*ν*_(*κh*) for the Euclidean distance lags *h*. The parameter *ν* measures the degree of smoothness and also the order of the modified Bessel function (when *ν* > 0) of second kind *K*_*ν*_ and finally *κ* > 0 is the scaling parameter with a range $\rho =(\sqrt{8\nu }/\kappa )$ where the spatial correlation is close to 0.1 for each *ν* (Lindgren et al., 2011; Rue et al., 2009).

### 2.2 Zero inflated Poisson and Binomial spatiotemporal models

Observational binary outcome data are commonly analysed using the logistic regression model, which has a logit linear predictor in the GLMMs canonical link structure. However this model has problems of instability especially with spatial random effects, which would be exacerbated due to zero inflation (Agarwal et al., 2002). In epidemiological cohort studies a relative risk is more preferred than an odds ratio as this caters for temporality and also a more intuitive measure of burden of morbidity or mortality (Barros and Hirakata, 2003; Fekedulegn et al., 2010). In light of this and in an endeavour to have better fitting models, two models that can handle zero inflation were employed. The two conditionally independent models fit were the zero inflated Poisson and zero inflated Binomial with a log and a logit canonical link functions respectively: ***y***_*t*_|***η***_*t*_, *θ*_*t*_ ∼ *ZIP*(***μ***_*t*_, *θ*_*t*_) and ***y***_*t*_|***η***_*t*_, *p*_*t*_, *θ*_*t*_ ∼ *ZIBin*(*n*_*t*_, *p*_*t*_, *θ*_*t*_).

The mortality outcome (count/binary) data ***y***_*t*_(*s*_*j*_) observed at the households in the Agincourt area are zero inflated and assumed to follow either a Poisson (count) or Binomial distribution (binary). We will occasionally drop the *s*_*j*_ for notational convenience in the rest of the article. We therefore resorted to the zero inflated models to cater for the imbalance due to many zeros. The model that takes care of zero inflation (*θ*_*t*_) can then be represented as:

(3) $$\pi (y_{t}|\eta_{t},\theta_{t})=\left\{\begin{array}{ll} \theta_{t}+(1-\theta_{t})\pi (0|\eta_{t},\theta_{t}) & \text{if}\;y_{t}=0\\ \left(1-\theta_{t})\pi (1|\eta_{t},\theta_{t}\right) & \text{if}\;y_{t}\neq 0 \end{array}\right)$$

With canonical links of the expected means: *g*(*E*(*y*_*t*_(.)) = log(***μ***_*t*_) = ***η***_*t*_ and *g*(*E*(*y*_*t*_(.)) = logit(*p*_*t*_) = ***η***_*t*_ with means are ***μ***_*t*_ = exp(***η***_*t*_) and *p*_*t*_ = (exp(***η***_*t*_)/(1 + exp(***η***_*t*_))) for the Poisson and Binomial distributions, respectively. A spatiotemporal canonical link (linear predictor) model can be expressed as

(4) $$\begin{array}{l} \eta_{t}(s_{j})=X_{t}(s_{j})\beta +f_{t}(s_{j})+\varepsilon_{t}(s_{j})\\ f_{t}(s_{j})=\phi \xi_{t-1}(s_{j})+\omega_{t}(s_{j}) \end{array}$$

where $\varepsilon_{t}(s_{j})∼N(0,\sigma_{\varepsilon }^{2}I_{m})$ with identity matrix *I*_*m*_ of dimension $m\times m,\omega_{t}∼N(0,\Sigma =\sigma_{\omega }^{2}\tilde{\Sigma })$ with a stationary AR(1) process ***ξ***_1_ ∼ *N*(***0***, *Σ*/(1 − *ϕ*^2^)) (Cameletti et al., 2012). The *Σ* is a dense GF *m* by *m* dimensional matrix from a Matérn distribution with scale and smoothness parameters *κ* and *ν* (which is fixed in all our computations) respectively. As the size of *m* increases computations become increasingly more difficult due to the “big m” as previously highlighted.

### 2.3 Solving the “big m” using the SPDEs to estimate the spatial random effects

**Fig. 1:**
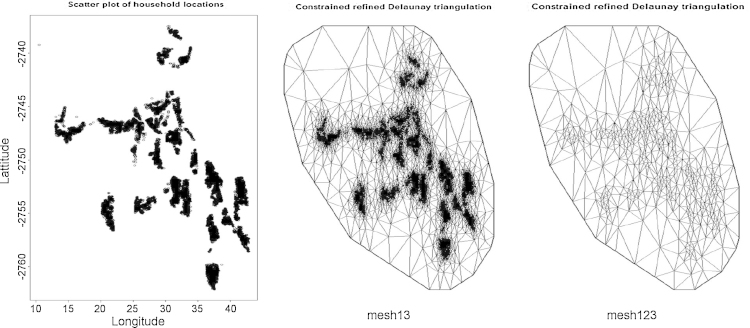
Agincourt original household locations (left), triangulation of all household (centre) and triangulation of households within 500 m (right).

To resolve the computational burden associated with the GF Matérn covariance function we used a technique that changes this to a GMRF proposed by Rue and Held (2005). Briefly the locations are converted into areal triangulations firstly by making them the initial triangle vertices before adding more vertices for proper triangulation which extends the grid and very useful for prediction. Fig. 1 shows how we employed triangulation to our data, the diagram on the right was made assuming households within 500 *m* were similar we fit 488 vertices and 938 triangles.

The SPDE technique redefines the Matérn field as a finite “representative” linear combination of basis functions on a triangulation of the locations (Cameletti et al., 2012). Hence for our spatiotemporal random effects in GMRF representation we get ${\tilde{\omega }}_{t}∼N(0,\Sigma =Q_{s}^{-1})$ and $\xi_{1}∼N(0,(Q_{s}^{-1}/(1-\phi^{2}))=Q_{T}^{-1})$ where *Q*_*s*_ is a sparse time-invariant precision matrix with dimension m* vertices from the triangulations. A joint spatiotemporal GMRF ${\tilde{f}}_{t}∼N(0,Q^{-1}={\left(Q_{T}⊗Q_{s}\right)}^{-1})$ whose precision matrix is the Kronecker product of the temporal and spatial precision matrices; is such that ${\tilde{f}}_{t}\approx Bf_{t}$ where the basis ***B*** is a sparse matrix with unit elements for matching triangle vertices and zero's elsewhere (Lindgren et al., 2011). Therefore Eq. (4) becomes ***η***_*t*_ = *X*_*t*_***β*** + ***B****f*_*t*_ + ***ɛ***_*t*_, where we let ***x***_*t*_ = {***β***, ***B***, *θ*_*t*_} be the vector of latent Gaussian fields and $\varphi_{t}=\{\sigma_{\omega }^{2},\phi ,\kappa ,\sigma_{\varepsilon }^{2}\}$ being a vector of unknown parameters. We can thus express our model into a hierarchical Gaussian latent variable fashion as follows, stage 1 – observational equation, stage 2 – latent Gaussian field and stage 3 – parameter model (Simpson et al., 2011):

(5) $$\begin{array}{l} \text{stage}\;1:\;y_{t}|x_{t},\varphi_{t}∼N(X_{t}\beta +Bf_{t};\sigma_{\varepsilon }^{2}I_{m})=N(\mu_{y_{t}}(\varphi_{t})=Ax_{t},Q_{y_{t}}^{-1}(\varphi_{t}))\\ \text{stage}\;2:\;x_{t}|\varphi_{t}∼N(\mu_{x_{t}}(\varphi_{t});Q_{x_{t}}^{-1}(\varphi_{t}))\\ \text{stage}\;3:\;\varphi_{t}∼\pi (\varphi_{t}) \end{array}$$

where the precision matrices $Q_{.}^{-1}(.)$ are either small enough (for easier multiple factorisation) or sparse (Simpson et al., 2011b). These models cover a wide range of models and are easily estimable using INLA as shown in the next subsection.

### 2.4 Bayesian inference using INLA

In accordance with the Bayesian paradigm we aim to find the posterior distribution of the processes and parameters updated by data (Wikle, 2003). This could be expressed as:

Probability   (process,   parameters|data)∝Likelihood   (data|process,   parameters)×Probability   (process|parameters)×Probability   (parameters)

Applying this expression to our model, letting $\Theta =\{\beta ,\phi ,\theta_{t},\sigma_{\omega }^{2},\kappa ,\sigma_{\varepsilon }^{2}\}$ denote the vector of all parameters and dropping the subscripts to present in vector form, ***ξ*** = {*ξ*_*t*_} and data ***y*** = {*y*_*t*_} (Cameletti et al., 2012; Rue et al., 2009), their joint posterior distribution is thus:

(6) π(Θ,ξ,η|y)∝π(y|Θ,ξ,η)π(η|ξ,Θ)π(ξ,Θ)

**Fig. 2:**
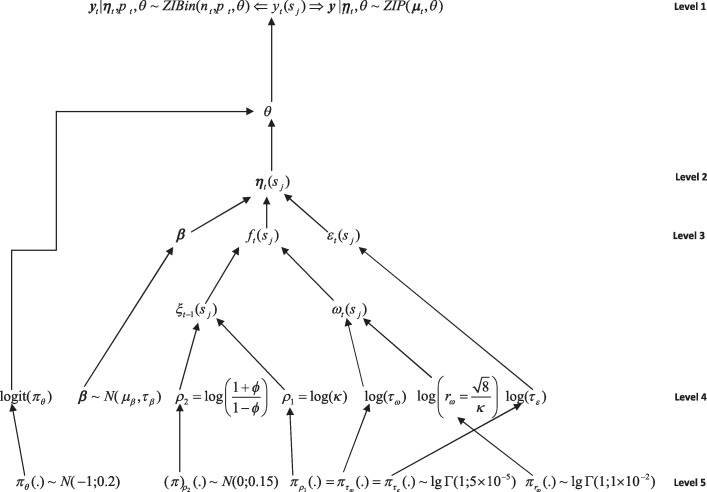
Hierarchical structure of a zero inflated spatiotemporal model fit using INLA.

Fig. 2 gives a simplified pictographical view, where level 1 are the data and assumed distributions, level 2 is a process, level 3 and 4 are parameters and level 5 gives the default hyper-parameters used in the INLA package.

The posterior marginals are required, standard (i), nested approximation (ii) and numerical integrations (iii) for latent fields ***x*** = {*β*, *f*, *θ*} and hyper-parameters $\varphi =\{\phi ,\sigma_{\omega }^{2},\kappa ,\sigma_{\varepsilon }^{2}\}$ respectively (Rue and Held, 2005; Rue et al., 2009):

(7) $$\begin{array}{ll} i)\pi (x,\varphi |y)=\int \pi (x_{i}|\varphi ,y)\pi (\varphi |y)\;d\varphi & \pi (\varphi_{j}|y)=\int \pi (\varphi |y)\;d\varphi_{-j}\\ ii)\tilde{\pi }(x,\varphi |y)=\int \tilde{\pi }(x_{i}|\varphi ,y)\tilde{\pi }(\varphi |y)\;d\varphi & \tilde{\pi }(\varphi_{j}|y)=\int \tilde{\pi }(\varphi |y)\;d\varphi_{-j}\\ iii)\tilde{\pi }(x,\varphi |y)=\sum_{k}\tilde{\pi }(x_{i}|\varphi_{k},y)\tilde{\pi }(\varphi_{k}|y)\Delta_{k} & \tilde{\pi }(\varphi_{j}|y)\propto \int {\left(\frac{\pi (x,\varphi |y)}{{\tilde{\pi }}_{G}(x|\varphi ,y)}\right|}_{x=x^{*}(\varphi )}\;d\varphi_{-j} \end{array}$$

Using this technique we aim to initially get the terms “nested” inside the integrand in Eq. (7ii) left hand side without integration (Simpson et al., 2011b). To do so we firstly estimate the marginal ${\tilde{\pi }}_{G}(x|\varphi ,y)$ which is a Gaussian approximation of ***x*** with mode ***x*** * (***φ***), for a given ***φ***. Secondly we estimate $\tilde{\pi }(x_{i}|\varphi ,y)=N(x_{i};\mu_{i}(\varphi ),\sigma_{i}^{2}(\varphi ))$ using either Gaussian or Laplace or a simplified Laplace approximations (Rue et al., 2009). We computed these marginals using the R package Integrated Nested Laplace Approximation (INLA) which uses the simplified Laplace approximations (Rue and Held, 2005; Rue et al., 2009; R-cran, 2010). The INLA procedure also enables easier spatial prediction since it computes posterior conditionals for the spatial random effects on all triangulation vertices including the extensions as shown in Fig. 1.

### 2.5 Model goodness of fit and convergence diagnostics

We assessed the accuracy of $\tilde{\pi }(\varphi |y)$ using the effective number of parameters, which can be approximated as the difference between the dimension of the normalised integral $\{\tilde{\pi }(\varphi |y)\}\;n$ and the trace of the product of the prior precision matrix and the posterior covariance (Spiegelhalter et al., 2002):

(8) $$p_{D}(\varphi )\approx n-tr\{Q(\varphi )Q*{\left(\varphi \right)}^{-1}\}$$

The deviance information criteria (DIC) was also used which is defined as the difference between twice the mean of the deviance and the deviance of the mean according to Spiegelhalter et al. (2002) and expressed as:

(9) $$D(x,\varphi )=-2\sum_{i}\text{log}\{\tilde{\pi }(y_{i}|x_{i},\varphi )\}+cons\tan t$$

Interpretations of these is quite straight forward the smaller the effective number of parameters the more parsimonious the model and the smaller the DIC the better the model fit, more-so most parsimonious is not always the best model.

## 3 Application

### 3.1 Rural South Africa Agincourt HDSS data, study design and ethics

The Agincourt health and demographic surveillance system (HDSS) site was set up in the Agincourt sub-district in 1992 due to its remote location, availability of several clinics and presence of Mozambican in-migrants (Tollman et al., 1999). By 2010, the Agincourt HDSS had a population of over 84,000 persons living in approximately 17,000 households scattered throughout 27 neighbouring villages. Cause of death data were obtained through verbal autopsies conducted on every recorded death (Clark et al., 2007). Interviews were conducted by trained field worker. This was done within 1 year after a death, with the closest caregiver of the deceased in their mother tongue. Cause of death was independently determined by two medical practitioners with the third as a tie breaker. Their consensus cause of death was classified according to the World Health Organization's International Classification of Diseases (ICD10) (Kahn et al., 2000). HIV/TB mortality in children was ascertained by the reported signs and symptoms, and in some instances this was verified through the mother's cause of death (Kahn et al., 2000). Over 90% of the HDSS households were geo-coded by 1992 and by 2010 all the households were geo-coded, thus enabling spatial analyses at household as well as village level. The study design was a retrospective cohort study covering households observed from the onset of the site to December 2010. The Agincourt HDSS site was granted ethical clearance by the University of the Witwatersrand's Committee for Research on Human Subjects (No. 960720). This work was also granted ethical clearance by the University of the Witwatersrand's Committee for Research on Human Subjects (M081145). Verbal informed consent was obtained when the census rounds were conducted and also when verbal autopsy data were collected from a close relative of the deceased.

### 3.2 Dependent and independent variables

The persons included in the study were all the children aged between 0 and under 10 years who lived or had lived in the Agincourt HDSS between January 1992 and December 2010. The independent variables used were: child's gender, birth-weight category, age category, slum (availability of water, electricity and toilet) and number of mother's antenatal clinic visits during pregnancy and year of observation. The household's latitude and longitude were used to construct the latent variables for the spatial random effects and year the AR(1) temporal effects. The dependent variable was death due to HIV and or tuberculosis (TB) determined by the WHOs ICD10 verbal autopsy codes A16-A19^1^ for HIV and B20-B24^2^ for TB. These data were extracted using Structured Query Language (SQL). Preliminary data analyses and data management were done using STATA 10.1 (StataCorp, 2007). The Bayesian analysis was done using an R software package called INLA (Rue et al., 2009; R-cran, 2010).

### 3.3 Descriptive statistics

**Fig. 3:**
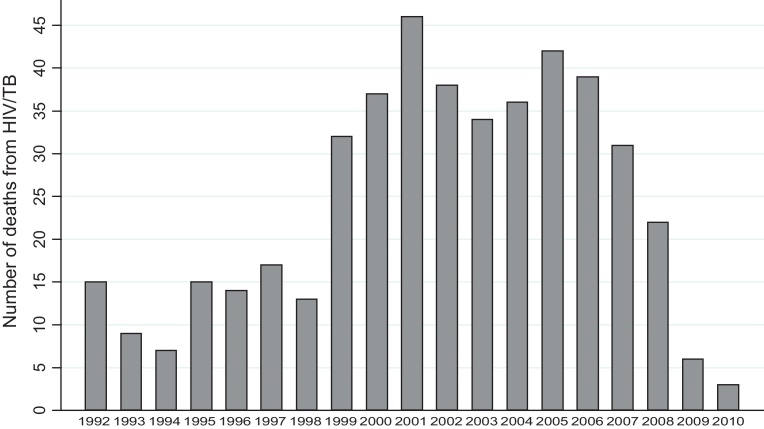
Year specific child deaths due to HIV/TB from 1992 to 2010.

There were similar proportions of boys and girls (50.3%) with an average age of 6.14 years (standard deviation of 3.38). Almost 9% of the births were low-weight, the majority (60.3%) of the children were only observed after their 5th birthday. Only 8.09% had access to all three: clean tap water, flush toilet inside house and electricity, which is expected in typical rural areas. Health check-ups during pregnancy averaged about 6 visits, with the majority (73%) still not going at all. A total of 456 deaths were HIV/AIDS (including HIV/tuberculosis) which was a small proportion of the population indicative of the presence of zero inflation. Mortality was moderate in the early to mid 1990s before gradually growing from 1999 and reaching a peak in 2001, remaining high for several years before gradually declining from 2007 as shown in Fig. 3.

### 3.4 Zero inflated Poisson and Binomial spatiotemporal modelling results

We performed univariate and multiple variable analyses using both the ZIP and ZIB models. Multiple variable models were fit systematically starting with one without random effects, spatial only, temporal only and finally spatiotemporal random effects. The R-codes using INLA are given in Appendix 1. From our results shown in Tables 1 and 2, our discussion will be centred on the ZIP spatiotemporal model which was the best fitting model since this had the lowest DIC (4532.31) and catered for spatiotemporal random effects, as later indicated in Section 3.5.

**Fig. 4:**
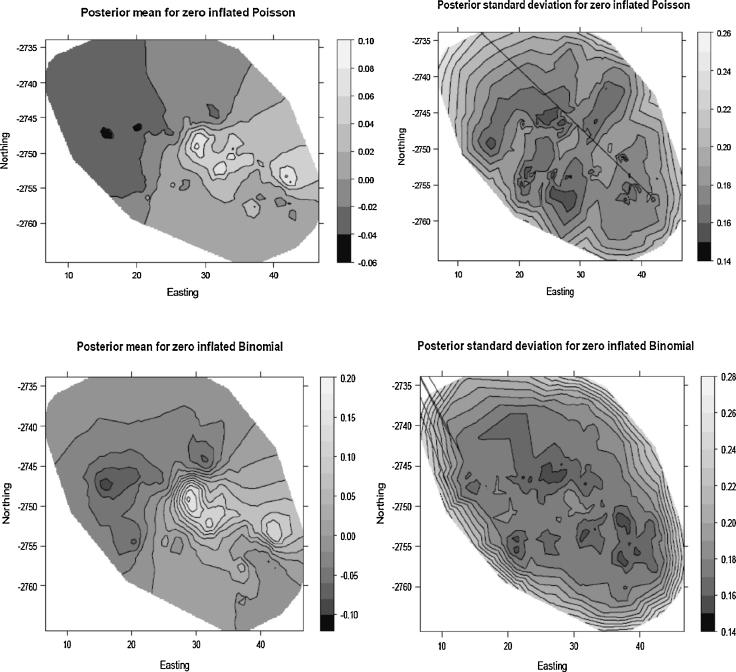
Posterior point estimates for ZIP (top) and ZIB (bottom) models.

Two variables consistently showed significant associations with child HIV/TB mortality, firstly the age category of the child, which showed a decrease in mortality with increase in age (Chi-Square *p*-value < 0.001). Those aged 1 to under 5 (127/19,619) were 82% {0.18(0.14; 0.22)} less likely to experience a death due to HIV/TB compared to those under 1(262/8580) and those over 5 and under10 (67/42,858) were 96%{0.04(0.03;0.05)} less likely to die from HIV/TB relative to the under 1 s keeping all other variables constant. Secondly the category of the birth weight was significantly associated with deaths due to HIV/TB, showing a decreasing trend with greater birth weight (Chi-Square *p*-value < 0.001). Those who had a birth weight of 3.5 kg and above (232/49,496) were 27% {0.73(0.53;0.99)} less likely to die due to HIV/TB relative to those who were born with a low birth weight of less than 2.5 kg (76/6329) independent of other risk factors. The only other significant protective factor was the number of antenatal clinic visits made by the mother; for every additional visit the mother attended the risk of losing the child from HIV/TB decreased by 4% {0.96(0.94;0.97)} keeping other variables constant as shown by the spatial only and no-random effects model. Those who had access to all three electricity, water and flush toilet (who in this area are the more affluent) experienced fewer deaths due to the HIV/TB compared to those who had none. We show the posterior marginal point estimates from the ZIP and ZIB models in Fig. 4 top and bottom respectively.

As shown in Fig. 4, the region with the greatest risk of child HIV/TB mortality is the central region with a trend towards the south-westerly. In the context of our findings, this is the region whose households have lower birth weights, greater under 1 mortality and having lower visits to the health facility compared to households in other regions. The lowest risk region is the top north-easterly with the ZIP model showing few clusters than the ZIB model. The standard error maps indicate the greatest errors on the peripherals which are also the regions where the extensions of the triangulation shown in Fig. 1. Also consistent with our results from Tables 1 and 2 there is a greater margin of error with the ZIB model as compared with the ZIP model.

### 3.5 Model assumptions, goodness of fit and convergence diagnostics

**Fig. 5:**
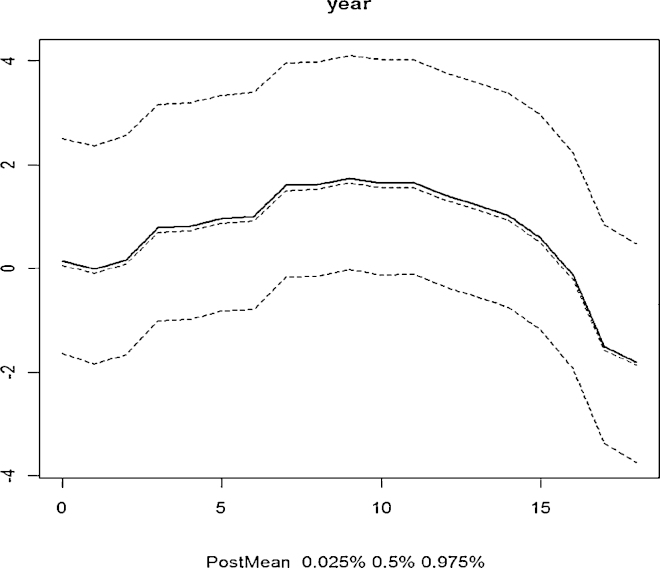
Posterior means (bold), medians (0.5% = 50th percentiles, middle), lower credible limits (0.025% = 2.5th percentiles) and upper credible limits (0.975% = 97.5th percentiles).

Using the final spatiotemporal models in both ZIB and ZIP we investigated the presence of zero inflation, temporal and spatial correlation. The null hypothesis for zero inflation was that the inflation parameter *θ*_*t*_ is zero (no zero inflation) our results in Tables 1 and 2 indicate significant presence of zero inflation of 0.04 and 0.42 for ZIP and ZIB respectively. Investigation of temporality was done by testing the *ϕ* coefficient of the first order autocorrelation model with null hypothesis that the process was not stationary |*ϕ*|>1. Both our models indicated significance presence of stationarity that is 0.86 and 0.87 for ZIP and ZIB respectively. Fig. 5 shows the posterior means plus 95% credible bands, time series plots from the modelling which resembles the observed data shown in Fig. 3. Lastly on checking model assumptions we investigated the presence of spatial effects by inspecting the significance of the components (*κ* and *τ*) of the Matérn covariance (see Tables 1 and 2). Also this was investigated with the classical Moran's indices on the spatial residuals testing the null hypothesis that there is zero spatial autocorrelation which from both tests yielded significant results, hence rejecting the null.

**Fig. 6:**
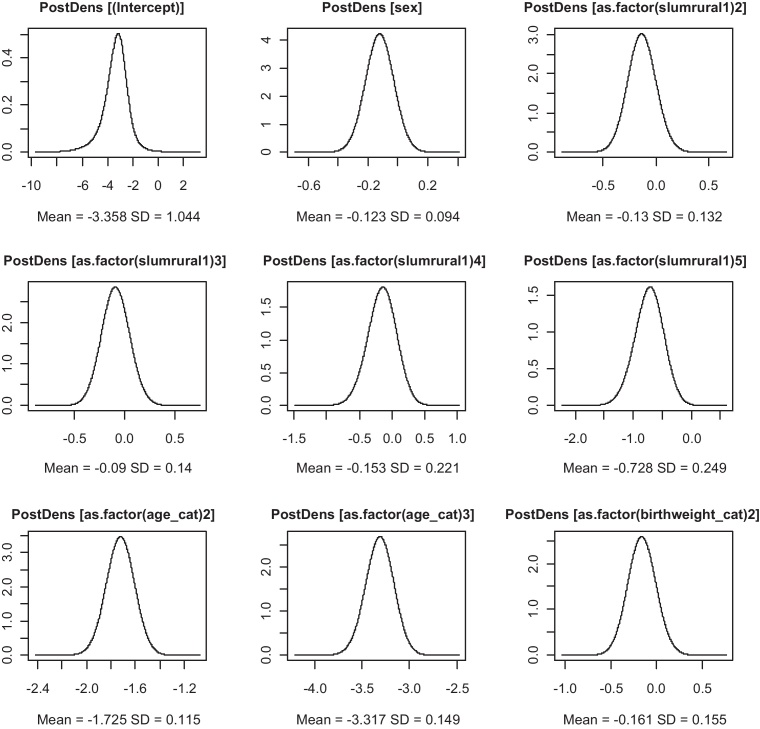
Posterior density plots of log-means for fixed effects of the ZIP spatiotemporal model.

**Fig. 7:**
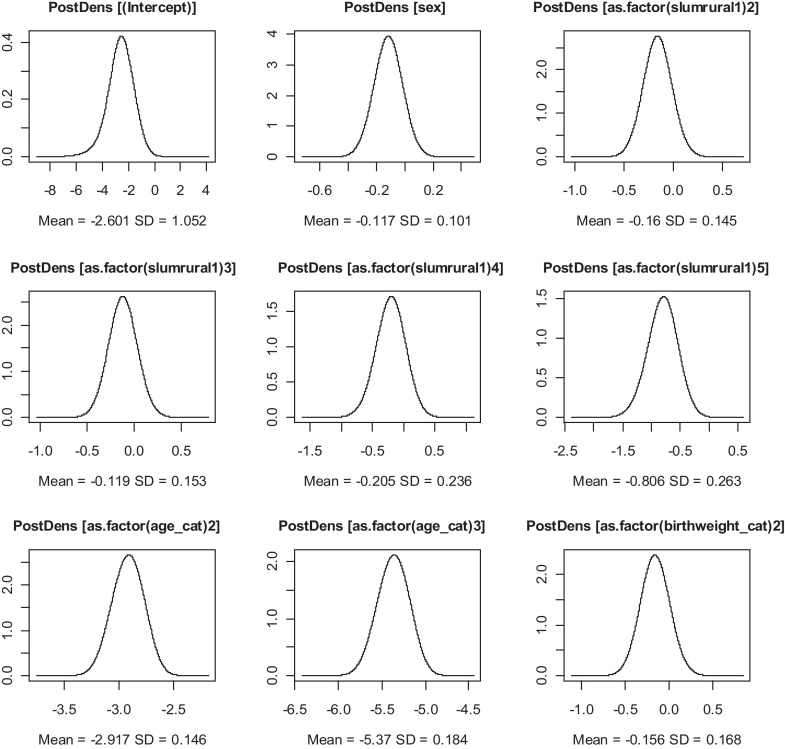
Posterior density plots of log-means for fixed effects of the ZIB spatiotemporal model.

We compared the two models the ZIB and ZIP and found that the latter fitted better in all the variants from the no-random effects model to the spatiotemporal as this yielded smaller DICs and model parsimony. Some of the parameter estimates were similar, except for the zero inflated parameter, age categories and birth weights. The ZIP model also showed more stable estimates and consistent results in the adjusted relative risks (adjusted RRs) compared to the ZIB and narrower credible intervals. As a way of showing the convergence pictographically for some parameter estimates we show the fixed effects logarithmic form posterior means density plots in Figs. 6 and 7.

The plots in Figs. 6 and 7 show that the parameters fitted well as GMRF since they are symmetrical about thus the mean equals the mean and mode.

## 4 Conclusions

The Public Health rational of our results is centred on lack of health seeking behaviour in rural areas. Interventions could target two groups of mothers (also caregivers) those not yet infected and those already infected. For both groups maternal attendance of health facility can minimize the risk of under one mortality and possibly giving birth to low-weight children. For those mothers’ already infected several options are there; before child birth (prevention of mother to child) and after birth (anti-retro-virals – ARV uptake) and also early treatment on ARVs for the parent to increase their longevity. A significant decline in mortality can already be observed in our cohort post 2007 the year when ARVs were started in the area. Poverty however still remains the greatest challenge in the developing countries and mostly interventions when available are very scarce. As such our results from spatiotemporal Bayesian modelling coupled with maps can be very handy in allocation of the limited resources of aid.

Modelling that controls for potential confounding is an add-on to epidemiological studies and gives strength to the estimates derived from such modelling procedures. Our modelling approach caters for potential spatial and temporal confounders. This approach was able also to cater for “large” zero inflated spatiotemporal data. The years 1992–2010 cover a span of 19 years (76%) of the United Nation's Millennium Development Goals (MDG) period of 25 years. Goal 4.1 aims at reducing child (under five) mortality by two thirds over that period (Black et al., 2003). Our results demonstrate such a significant decline in mortality due to HIV/TB.

A major limitation in developing nations is lack of reliable and user friendly analyses software, which was addressed by using INLA a package available on the public domain. Zero inflated Poisson (ZIP) and zero inflated Negative Binomial (ZINB) models were used for log link function linear predictors and also catered for spatial random effects (Lambart, 1992; Ridout et al., 2001). It has also been noted from other studies that the log link function linear predictor models were more stable than logit link function models when they contained a spatial component. This finding was also confirmed in our study as we observed that models from the exponential family with a log-linear predictor were more stable and able to converge better than the logit-link function model. This motivated us to treat the outcome as a discrete measure as opposed to binary mostly used for logistic regression (Agarwal et al., 2002). We demonstrated that the “big m” can be resolved with great ease for Public Health mortality hierarchical structures with the aid of SPDEs and INLA.

## Contributions

EM drafted the manuscript and did the statistical analysis. KK provided critical intellectual content and TFC and PV the statistical content. KK was also responsible for the design and quality of the Agincourt verbal autopsy data. All authors read and approved the final manuscript.

## Conflict of interest

The authors declare that they have no competing interests.

## Figures and Tables

**Table:** 





**Table 1:** Univariate and multiple regression results models using zero inflated Poisson adjusting for spatiotemporal random effects.

Variable	Summary, *n* (%) or mean ± SD	Univariate results, RR (95% credible interval)	Non-spatial multiple variable model, adjusted RR (95% credible interval)	Temporal multiple variable model, adjusted RR (95% credible interval)	Spatial multiple variable model, adjusted RR (95% credible interval)	Spatiotemporal multiple variable model, adjusted RR (95% credible interval)
**Sex**						
Male	35,317(49.70)	1.00				
Female	35,740(50.30)	0.89(0.74;1.07)				
**Birth weight category**						
Low weight	6,320(8.89)	1.00	1.00	1.00	1.00	1.00
Moderate weight	15,241(21.45)	0.81(0.61;1.07)	0.70*(0.53;0.93)	0.85(0.63;1.16)	0.71*(0.53;0.93)	0.85(0.63;1.16)
High weight	49,496(69.66)	0.39*(0.30;0.51)	0.57*(0.42;0.76)	0.73*(0.53;0.99)	0.58*(0.43;0.77)	0.73*(0.53;0.99)
**Age category**						
0–1 years	8,580(12.07)	1.00	1.00	1.00	1.00	1.00
1–5 years	19,619(27.61)	0.21*(0.17;0.26)	0.20*(0.16;0.25)	0.18*(0.23;0.64)	0.20*(0.16;0.25)	0.18*(0.14;0.22)
5–9 years	42,858(60.31)	0.05*(0.04;0.07)	0.04*(0.03;0.05)	0.04*(0.05;0.54)	0.04*(0.03;0.05)	0.04*(0.03;0.05)
**Slum (electricity, water and toilet)**						
None of the three	10,051(14.14)	1.00	1.00		1.00	
At least one	27,329(38.46)	0.78(0.60;1.01)	0.79(0.61;1.02)		0.77(0.60;1.00)	
At least two	21,871(30.78)	0.74*(0.56;0.97)	0.73*(0.56;0.96)		0.71*(0.54;0.93)	
All three	5,748(8.09)	0.57*(0.37;0.86)	0.55*(0.35;0.83)		0.53*(0.34;0.81)	
**Antenatal clinic visits**	6.00(±1.50)	0.98*(0.97;0.99)	0.98*(0.97;0.99)	0.96*(0.94;0.97)	0.98*(0.96;0.99)	0.96*(0.94;0.97)
Zero inflation parameter			0.06*(0.002;0.25)	0.05*(0.003;0.18)	0.07*(0.003;0.28)	0.04*(0.002;0.15)
Precision for year				0.91*(0.23;2.19)		0.86*(0.16;2.32)
Rho for year				0.88*(0.72;0.97)		0.89*(0.70;0.98)
Kappa					1.98*(1.09;3.66)	1.55*(1.41;1.63)
Tau					1.37*(0.18;2.600	2.31*(1.19;9.08)
Moran's Indexes: Observed(Expected ± standard deviation)						0.32*(2.1 ± *3.33* × *10*^*−3*^*)*
Effective number of parameters			*12.05(0.05)*	*25.61(1.10)*	*23.18(5.04)*	*29.16(6.33)*
DIC			*4855.31*	*4532.69*	*4846.13*	*4532.31*

* Statistical significance at the 5% level.

**Table 2:** Multiple regression results of four models using zero inflated Binomial adjusting for spatiotemporal random effects.

Variable	Non-spatial multiple variable model, adjusted OR (95% credible interval)	Temporal multiple variable model, adjusted OR (95% credible interval)	Spatial multiple variable mode, adjusted OR (95% credible interval)	Spatiotemporal multiple variable model, adjusted OR (95% credible interval)
**Sex**				
Male				
Female				
**Birth weight category**				
Low weight	1.00	1.00	1.00	1.00
Moderate weight	0.70*(0.52;0.92)	0.86(0.62;1.20)	0.70*(0.52;0.93)	0.005*(0.003;0.07)
High weight	0.62*(0.46;0.83)	0.67*(0.48;0.95)	0.63*(0.47;0.85)	0.05*(0.007;0.04)
**Age category**				
0–1 years	1.00	1.00	1.00	1.00
1–5 years	0.07*(0.05;0.09)	0.05*(0.04;90.07)	0.07*(0.06;0.09)	0.18*(0.14;0.22)
5–9 years	0.006*(0.004;0.008)	0.005*(0.003;0.95)	0.006*(0.004;0.008)	0.04*(0.03;0.05)
**Slum (electricity, water and toilet)**				
None of the three	1.00		1.00	
At least one	0.77(0.60;1.01)		0.76*(0.58;0.99)	
At least two	0.71*(0.54;0.95)		0.69*(0.52;0.93)	
All three	0.52*(0.34;0.81)		0.51*(0.32;0.78)	
**Antenatal clinic visits**		0.94*(0.92;0.96)	0.97*(0.95;0.98)	0.94*(0.93;0.96)
Zero inflation parameter	0.22*(0.042;0.66)	0.43*(0.19;0.79)	0.15*(0.031;0.45)	0.42*(0.18;0.67)
Precision for year		0.78*(0.17;1.96)		0.80*(0.20;1.93
Rho for year		0.87*(0.67;0.97)		0.87*(0.69;0.97)
Kappa			1.98*(1.14;3.55)	1.54*(1.41;1.62)
Tau			1.37*(0.27;2.54)	2.23*(1.22;6.91)
Moran's Indexes: Observed(Expected ± standard deviation)				0.30*(2.1 ± *3.33* × *10*^*−3*^*)*
Effective number of parameters	*11.10(0.080)*	*24.58(1.03)*	*22.97(5.09)*	*31.12(5.18)*
DIC	*5179.43*	*4754.60*	*5180.50*	*4803.93*

* Statistical significance at the 5% level.

## Footnotes

1 A16 = respiratory tuberculosis; not confirmed bacteriologically or histologically; A17 = tuberculosis of nervous system; A18 = tuberculosis of other organs; A19 = miliary tuberculosis.

2 B20 = human immunodeficiency virus (HIV) disease resulting in infectious and parasitic diseases; B21 = human immunodeficiency virus (HIV) disease resulting in malignant neoplasms; B22 = human immunodeficiency virus (HIV) disease resulting in other specified diseases; B23 = human immunodeficiency virus (HIV) disease resulting in other conditions; B24 = unspecified human immunodeficiency virus (HIV) disease.
